# Factors associated with HIV-positive status among men who have sex with men in Guinea in 2022

**DOI:** 10.3389/fpubh.2025.1633546

**Published:** 2025-08-28

**Authors:** Mamadou Samba Dioum, Septime Hessou, Tamba Mina Millimouno, Fassou Mathias Grovogui, Delphin Kolié, Thierno Saidou Diallo, Elhadji Mamadou Dioukhane, Elhadj Marouf Diallo, Alioune Camara, Thierno Mamadou Tounkara, Alexandre Delamou, Fodé Bangaly Sako

**Affiliations:** ^1^Plan International, Conakry, Guinea; ^2^Centre d’Excellence d’Afrique pour la Prévention et le Contrôle des Maladies Transmissibles, Faculté des Sciences et Techniques de la Santé, Université Gamal Nasser de Conakry, Conakry, Guinea; ^3^Centre National de Formation et de Recherche en Santé Rurale de Maferinyah, Forécariah, Guinea; ^4^Programme National de Lutte contre le VIH/Sida et les Hépatites, Conakry, Guinea; ^5^Plan International Canada, Toronto, ON, Canada; ^6^Programme National de Lutte contre le Paludisme, Conakry, Guinea; ^7^Service de Dermatologie-Vénérologie, Centre Hospitalo-Universitaire de Donka, Conakry, Guinea; ^8^Direction Nationale de l’Epidémiologie et de la Lutte contre la Maladie, Ministère de la Santé, Conakry, Guinea

**Keywords:** HIV positive, associated factor, MSM (men having sex with men), Guinea (Conakry), 2022

## Abstract

Introduction/Context Men who have sex with men (MSM) continue to carry a disproportionate burden of HIV in West and Central Africa, where stigma, criminalization, and legal restrictions hinder access to essential prevention and treatment services. In Guinea, data on HIV-related risk factors among MSM remain scarce. Objective This study aimed to estimate the prevalence of HIV and identify factors associated with HIV seropositivity among MSM in Guinea using nationally representative data. Methods We conducted a cross-sectional analysis of the 2022 Integrated Bio-Behavioral Surveillance (IBBS) survey data, involving 1,692 MSM aged 15 years and older from eight administrative regions. Participants were recruited through respondent-driven sampling, completed standardized behavioral questionnaires, and underwent HIV testing. Univariate and multivariate logistic regression analyses were performed to identify factors associated with HIV seropositivity. Results The overall HIV prevalence among MSM was 9.4%, with higher rates in Conakry (13.4%) and N’Zérékoré (10.5%). After adjusting for confounders, three factors were significantly associated with higher odds of HIV seropositivity: being aged 25 years or older (adjusted odds ratio [aOR] = 1.78; 95% CI: 1.25–2.54), reporting STI symptoms in the past 6 months (aOR = 2.49; 95% CI: 1.61–3.84), and lacking knowledge of a regular partner’s HIV status (aOR = 1.60; 95% CI: 1.11–2.31). Conclusion These findings highlight the need for targeted, stigma-free HIV interventions for MSM in Guinea. Strategies should emphasize STI screening, support partner disclosure, and address structural barriers to care.

## Introduction

Men who have sex with men (MSM) are defined as individuals assigned male at birth who engage in sexual activity with other men, regardless of whether they also have sex with women or identify as bisexual or gay in personal or social contexts (1).

According to the Joint United Nations Program on HIV/AIDS (UNAIDS), MSM and other key populations accounted for 74% of all new HIV infections globally in 2021. The risk of acquiring HIV among MSM was estimated to be 28 times higher than that of other men in the general population. In 2022, the global median HIV prevalence among adults aged 15 to 49 years was 0.7%, compared to 7.7% among MSM (2, 3).

Globally, stigma, discrimination, and human rights violations—often institutionalized through punitive laws create substantial barriers to conducting effective HIV-related research and delivering tailored policies and health services for MSM (4). These barriers are particularly acute in sub-Saharan Africa, where many countries criminalize same-sex sexual behavior, and where HIV continues to disproportionately affect MSM (5, 6).

In Guinea, the 2018 Demographic and Health Survey estimated that 1.3% of men were living with HIV. An analysis of HIV epidemic dynamics and cascade indicators drawing on routine health data, surveys, and research conducted between 2012 and 2017 highlighted a concentrated epidemic among key and vulnerable populations, particularly MSM, who had an HIV seroprevalence of 11.4% (6).

In 2017, HIV prevalence among MSM in Guinea was three times higher in those aged 25 years and older compared to those aged 18–24 years (7.5%). MSM identifying as receptive partners had a substantially higher HIV prevalence (25.8%) compared to insertive (6.6%) and versatile (7.8%) individuals. Regional disparities were also noted, with prevalence rates among MSM exceeding the national average in N’Zérékoré (17.1%), Kankan (14.6%), and Boké (12.1%) (6).

Despite notable progress in recent years toward achieving the UNAIDS 95–95-95 targets among key populations especially in terms of resource mobilization, service coverage, and the provision of MSM-specific health services HIV prevalence remains disproportionately high among MSM in Guinea. However, the determinants of HIV infection within this group remain underexplored and poorly documented.

This evidence gap hinders the development of tailored, evidence-based interventions. Addressing it through dedicated research is essential to inform the design of targeted HIV prevention and care strategies that respond to the specific needs of MSM in Guinea.

The aim of this study was to analyze the factors associated with HIV seropositivity among MSM in Guinea, based on data from the 2022 national Integrated Biological and Behavioral Survey (IBBS).

## Methods

### Study design and sampling procedures

This study is a secondary analysis of data from the 2022 Integrated Biological and Behavioral Survey (IBBS) conducted among key populations in Guinea. The IBBS employed a cross-sectional design and utilized respondent-driven sampling (RDS), a peer-referral methodology suitable for hard-to-reach populations such as MSM. Recruitment began with a diverse group of selected “seeds” based on region, age, and network size, who then referred eligible peers using uniquely coded coupons. Participants were eligible if they were assigned male at birth, aged 15 years or older, reported having had sex with another man in the past 12 months, and provided informed consent.

### Setting

#### General context

The Republic of Guinea is located in West Africa and had an estimated population of 13,261,638 in 2022, with 64% residing primarily in rural areas (7).

Guinea’s health system is organized into three levels: central (Ministry of Health), regional (eight regional health inspectorates), and district (38 health districts). The country’s epidemiological profile remains dominated by communicable diseases, particularly STIs, HIV/AIDS (8).

##### Specific context: socio-cultural and legal environment

Like most West African countries, Guinea is a society where prostitution, homosexuality, and all forms of sexual minorities are not socially, culturally, or legally accepted or tolerated. These socio-cultural pressures may hinder access to preventive and curative care and the protection of the rights of MSM. They also contribute to the social rejection and exposure of MSM to violence.

Legally, Article 274 of the new Guinean Penal Code states: “Any act of indecency or against nature committed with a person of the same sex is punishable by imprisonment from 6 months to 3 years and a fine of 500,000 to 1,000,000 Guinean francs” (9).

However, Law L/025, enacted in 2005, contains provisions for the protection of people living with HIV. In 2009, to improve the implementation conditions of HIV prevention, testing, treatment, and care services, Decree No. 056 was enacted in Guinea, related to the Prevention, Care, and Control of HIV/AIDS (Presidency of the Republic, 2009).

This decree defines sexual minorities as individuals whose sexual orientation deviates from heterosexuality, including MSM, lesbians, bisexuals, and intersex persons, regardless of whether they self-identify as such (10).

### Intervention package for MSM in Guinea

The national health policy promotes universal access to quality care without geographic, economic, or socio-cultural barriers, based on complementarity between the public, private, associative, and community sectors. Similarly, the national gender policy adopted in 2011 aims to ensure equitable access to basic social services, including health care for men, women, boys, and girls (11).

In Guinea, the prevention package provided to MSM includes: peer-led educational talks at MSM gathering sites; awareness and education sessions via social networks conducted by peer educators through key population community centers in Conakry and Kindia; distribution of health commodities (male condoms and lubricants); referral for HIV and STI testing to adapted service centers; use of Medical Mobile Units and Community Clinics; provision of HIV self-testing kits at MSM hotspots; and treatment for STIs and HIV/AIDS (12).

This study was conducted across all eight health regions of the country (Conakry, Kindia, Boké, Mamou, Labé, Kankan, Faranah, and N’Zérékoré), each of which has a dedicated service center for key populations, including MSM.

### Study population

All MSM aged 15 years or older or MSM, regardless of whether they also have sex with women or identify as bisexual or gay who consented to participate and underwent HIV testing were included in the study.

### Study variables and data sources

The sociodemographic variables collected included administrative region, age, and level of education. Sexual behavior variables comprised sexual orientation, primary sexual role (insertive, receptive, or versatile), multiple male sexual partnerships, use of illicit drugs, knowledge of a regular partner’s HIV status, and presence of symptoms suggestive of sexually transmitted infections (STIs).

The main outcome variable was HIV serostatus (positive or negative), determined using the national HIV testing algorithm based on three rapid diagnostic tests: Determine™, Bioline™, and Multisure™.

Data were extracted from the national database of the 2022 Integrated Biological and Behavioral Survey (IBBS). Data collection was conducted across eight administrative regions—Conakry, Kindia, Boké, Mamou, Labé, Kankan, Faranah, and N’Zérékoré—using standardized behavioral questionnaires and HIV testing protocols aligned with international IBBS guidelines. All field teams were thoroughly trained, and the survey procedures strictly followed the national bio-behavioral surveillance protocol, approved by the Ministry of Health^1^.

### Data analysis

The dataset was exported from Excel and analyzed using SPSS version 26. Descriptive statistics were presented as means with standard deviations (SD), medians with interquartile ranges (IQR), or proportions with corresponding 95% confidence intervals (CI), as appropriate. The primary outcome was HIV seropositivity.

Bivariate analyses were performed to compare characteristics between HIV-positive and HIV-negative MSM. Categorical variables were analyzed using the Chi-square test or Fisher’s exact test, while continuous variables were compared using the Student’s t-test. Analyses were stratified by region and included key sociodemographic, behavioral, and biological variables. All statistical tests were two-sided, with a significance level set at *p* ≤ 0.05.

Variables with a *p*-value ≤ 0.20 in the bivariate analysis were considered for inclusion in a multivariable logistic regression model to identify factors independently associated with HIV seropositivity. A forward stepwise selection method was used to build the final model. Multicollinearity was assessed using the Variance Inflation Factor (VIF), and variables with VIF values >10 were excluded. The goodness-of-fit of the final model was evaluated using the Hosmer–Lemeshow test and pseudo-R^2^ measures. Adjusted odds ratios (aOR) with 95% confidence intervals were reported.

### Ethical considerations

The study protocol received approval from the National Health Research Ethics Committee (Approval No. 004/CNERS 2022).

All individual data used for this secondary analysis were anonymized, and confidentiality was strictly maintained.

## Results

**Figure 1:**
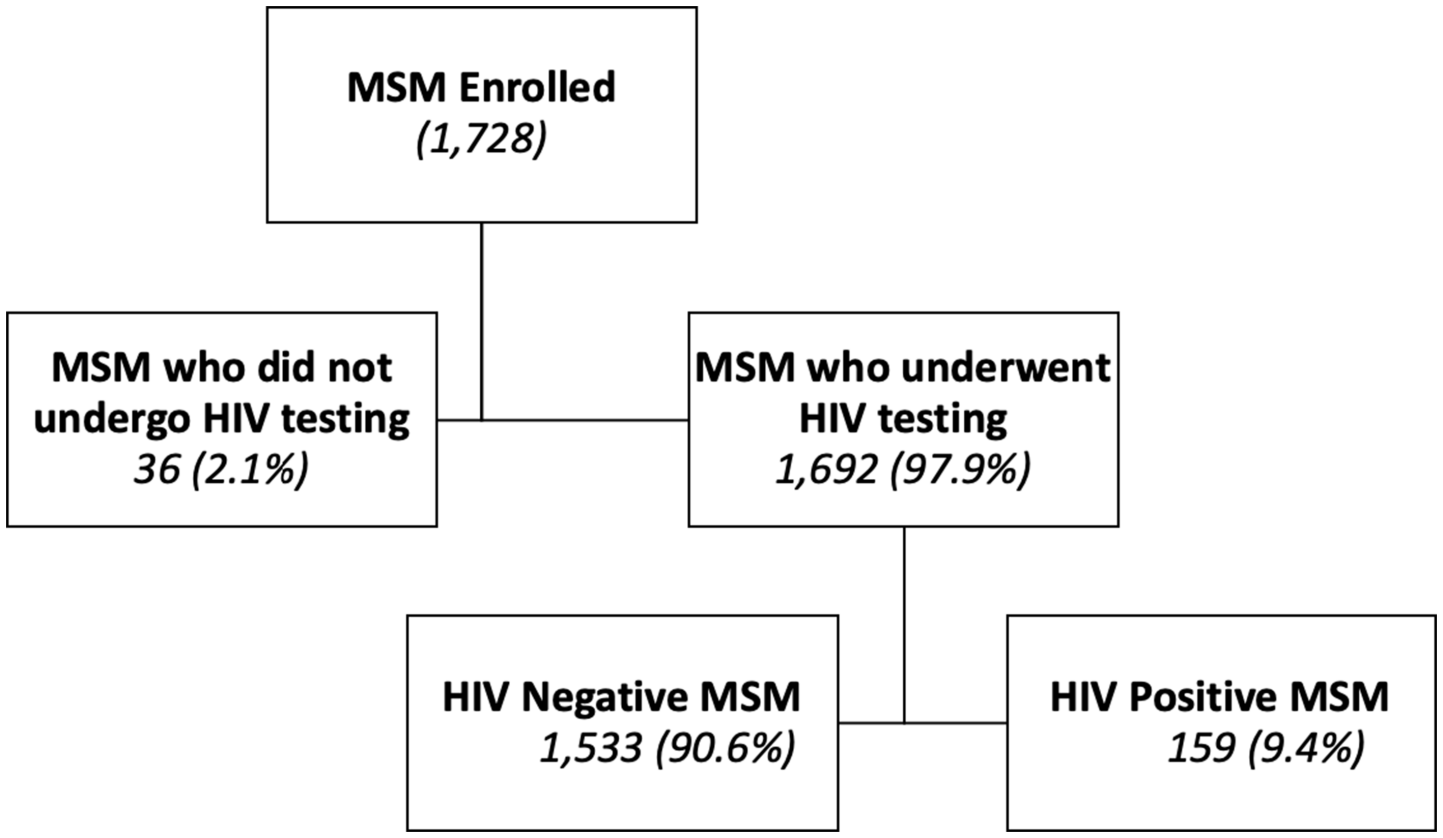
Flow of inclusion of MSM during the ESCOMB study in 2022 in Guinea.

**Figure 2:**
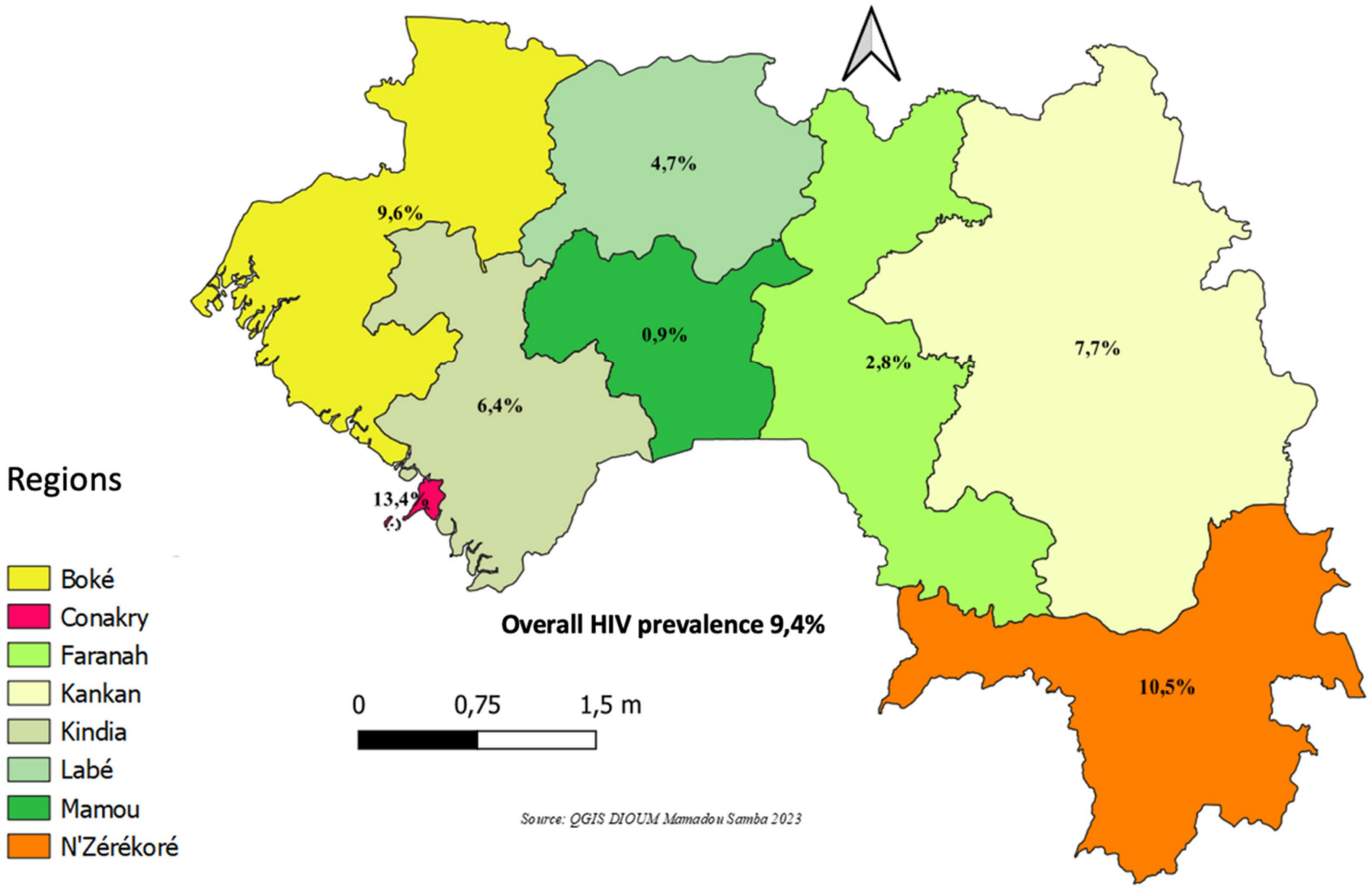
HIV prevalence among men who have sex with men (MSM) by administrative region, Guinea, 2022 (*N* = 1,692).

Among the 1,728 MSM surveyed, 1,692 (97.9%) underwent HIV testing and were included in the analysis. The median age of participants was 23 years (interquartile range [IQR]: 23–26), and 66.5% were aged 15–24 years (Figures 1, 2). Approximatively half of the participants resided in Conakry (44.4%), and 44.0% had attained secondary education. Sexual orientation was evenly distributed between bisexual (48.7%) and homosexual (48.0%) identities, while the most commonly reported sexual role was insertive (58.2%). A summary of sociodemographic and behavioral characteristics is provided in Table 1.

**Table 1:** Characteristics of the sample (*N* = 1,692).

Characteristics	Categories	Frequency	Proportion (%)
Administrative Region	Boké	135	8.0
	Conakry	751	44.4
	Faranah	141	8.3
	Kankan	182	10.8
	Kindia	203	12.0
	Labé	85	5.0
	Mamou	109	6.4
	N’Zérékoré	86	5.1
Age Group	15–24 years	1,125	66.5
	≥ 25 years	562	33.2
Educational Level	No formal education	252	14.9
	Primary education	40	2.4
	Secondary education	744	44.0
	Higher education	626	37.0
	Other	30	1.8
Sexual Orientation	Homosexual	813	48.0
	Bisexual	866	48.7
	No response	13	0.8
Main Sexual Role	Insertive	984	58.2
	Receptive	583	34.5
	Versatile	121	7.2
	No response	4	0.2
Multiple Sexual Partnerships	Yes	1,092	64.5
	No	596	35.2
	No response	4	0.2
Practice of Fellatio	Yes	441	26.1
	No	1,246	73.6
	No response	5	0.3
Paid Anal Intercourse	Yes	252	14.9
	No	1,436	84.9
	No response	4	0.2
Group Sexual Intercourse	Yes	95	5.6
	No	1,593	94.1
	No response	4	0.2
Presence of STI Symptoms	Yes	1,135	67.1
	No	404	23.9
	No response / Do not know	153	9.0
Sexual Intercourse Under Influence of Alcohol	Yes	260	15.4
	No	1,398	82.6
	No response / Do not know	34	2.0
Sexual Intercourse Under Influence of Drugs	Yes	78	4.6
	No	378	22.3
	No response / Do not know	1,236	73.0
Knowledge of Regular Partner’s HIV Status	Yes	727	43.0
	No	777	45.9
	No response	188	11.1

Median age: 23 years (IQR: 20–26).

The overall HIV prevalence was 9.4%. Prevalence was highest in the administrative regions of Conakry (13.4%), N’Zérékoré (10.5%), and Boké (9.6%). HIV prevalence was also elevated among participants aged ≥25 years (11.9%), those with primary education (17.5%), those identifying a receptive sexual role (15.1%), those reporting symptoms of sexually transmitted infections (STIs) (11.3%), those who had engaged in oral sex (15.4%) or group sex (12.6%), and those unaware of their regular partner’s HIV status (11.7%) (Table 2).

**Table 2:** HIV prevalence according to the characteristics of MSM en in Guinea, 2022 (*N* = 1,692).

Characteristics	Categories	HIV− n (%)	HIV+ n (%)	*p*-value
Region	Boké	122 (90.4%)	13 (9.6%)	0.108
	Conakry	650 (86.6%)	101 (13.4%)	
	Faranah	137 (97.2%)	4 (2.8%)	
	Kankan	168 (92.3%)	14 (7.7%)	
	Kindia	190 (93.6%)	13 (6.4%)	
	Labé	81 (95.3%)	4 (4.7%)	
	Mamou	108 (99.1%)	1 (0.9%)	
	N’Zérékoré	77 (89.5%)	9 (10.5%)	
Age (years) (*N* = 1,687)	15–24	1,035 (92.0%)	90 (8.0%)	0.002
	≥25	495 (88.1%)	67 (11.9%)	
Educational Level	No formal education	230 (91.3%)	22 (8.7%)	0.500
	Primary	33 (82.5%)	7 (17.5%)	
	Secondary	692 (93.0%)	52 (7.0%)	
	Higher education	552 (88.2%)	74 (11.8%)	
	Other	26 (86.7%)	4 (13.3%)	
Sexual Orientation (*N* = 1,679)	Homosexual	711 (87.5%)	102 (12.5%)	0.001
	Bisexual	811 (93.6%)	55 (6.4%)	
Main Sexual Role (*N* = 1,688)	Insertive	928 (94.3%)	56 (5.7%)	<0.001
	Receptive	495 (84.9%)	88 (15.1%)	
	Versatile	108 (89.3%)	13 (10.7%)	
Multiple Sexual Partnerships (*N* = 1,688)	Yes	995 (91.1%)	97 (8.9%)	0.423
	No	536 (89.9%)	60 (10.1%)	
Presence of STI Symptoms	Yes	1,007 (88.7%)	128 (11.3%)	<0.001
	No	383 (94.8%)	21 (5.2%)	
Practice of Fellatio (*N* = 1,687)	Yes	373 (84.6%)	68 (15.4%)	0.081
	No	1,157 (92.9%)	89 (7.1%)	
Group Sexual Intercourse (*N* = 1,688)	Yes	83 (87.4%)	12 (12.6%)	0.250
	No	1,448 (90.9%)	145 (9.1%)	
Paid Anal Intercourse (*N* = 1,688)	Yes	229 (90.9%)	23 (9.1%)	0.900
	No	1,302 (90.7%)	134 (9.3%)	
Knowledge of Regular Partner’s HIV Status (*N* = 1,504)	Yes	673 (92.6%)	54 (7.4%)	0.005
	No	686 (88.3%)	91 (11.7%)	
Sex Under Influence of Alcohol (*N* = 1,658)	Yes	236 (90.8%)	24 (9.2%)	0.915
	No	1,266 (90.6%)	132 (9.4%)	
Sex Under Influence of Drugs (*N* = 456)	Yes	72 (92.3%)	6 (7.7%)	0.520
	No	340 (89.9%)	38 (10.1%)	

HIV−: HIV negative serological status; HIV+: HIV positive serological status. p-values presented correspond to the overall comparison across categories of each variable, using the chi-square or Fisher’s exact test.

### Univariate analysis

Univariate logistic regression identified several variables significantly associated with HIV seropositivity (Table 2). Higher odds of HIV infection were observed among:

Participants aged ≥25 years (OR: 1.85; 95% CI: 1.30–2.63; *p* = 0.001),

Those identifying a receptive sexual role (OR: 2.80; 95% CI: 1.94–4.06; *p* < 0.001),

Participants reporting STI symptoms in the past 6 months (OR: 2.21; 95% CI: 1.51–3.23; p < 0.001),

Those unaware of their regular partner’s HIV status (OR: 1.60; 95% CI: 1.17–2.20; *p* = 0.003),

Sexual orientation also showed significant differences, with bisexual MSM having lower odds of infection compared to homosexual MSM (OR: 0.61; 95% CI: 0.45–0.84; *p* = 0.002).

### Multivariable analysis

All variables with a *p*-value ≤ 0.20 in the bivariate analysis were considered for inclusion in the multivariable logistic regression model. Following adjustment for potential confounders (Table 3), three variables remained independently and significantly associated with HIV seropositivity:

**Table 3:** Factors associated with HIV-positive status among MSM in Guinea, 2022.

Characteristics	Categories	*p*-value	Odds Ratio	Confidence Interval (Min–Max)
Age	15–24 years (ref.)			
	≥25 years	0.001	1.78	1.247–2.539
Presence of STI symptoms	No (ref.)			
	Yes	<0.001	2.49	1.608–3.839
Knowledge of regular partner’s HIV status	Yes (ref.)			
	No	0.012	1.60	1.110–2.306

Participants aged ≥25 years had significantly higher odds of being HIV seropositive compared to those aged 15–24 years (adjusted odds ratio [aOR]: 1.78; 95% confidence interval [CI]: 1.25–2.54; *p* = 0.001),

Self-reported STI symptoms within the past 6 months were associated with a markedly increased likelihood of HIV seropositivity (aOR: 2.49; 95% CI: 1.61–3.84; *p* < 0.001).

Not being aware of the HIV status of a regular sexual partner was independently associated with significantly higher odds of HIV seropositivity (adjusted odds ratio [aOR]: 1.60; 95% CI: 1.11–2.31; *p* = 0.012).

Although other sociodemographic and behavioral variables were initially examined in the bivariate analysis, they were not retained in the final model due to lack of statistical significance or multicollinearity. Specifically, sexual role, while showing a significant crude association, was excluded due to multicollinearity (variance inflation factor >10), which undermined model reliability. Educational attainment did not meet the predefined threshold for inclusion (*p* > 0.20), and data related to condom use exhibited a high proportion of missing or inconsistent responses, limiting its analytic utility.

The final model was therefore specified to ensure statistical robustness, parsimony, and internal validity, and should be interpreted in light of the inherent limitations of cross-sectional data, including potential residual confounding.

## Discussion

This study, which analyzed the factors associated with HIV infection among MSM in Guinea, found an overall HIV prevalence of 9.4%. This rate is approximatively seven times higher than the national HIV prevalence among young Guinean men in the general population (1.6%), highlighting the heightened vulnerability of MSM to HIV infection (13).

Although HIV prevalence among MSM has shown a slight decline from 11.4% in the 2017 IBBS (14); the current rate remains approximately 1.3 times higher than the average reported among MSM in West and Central Africa (15). Compared to neighboring countries, the prevalence observed in this study is lower than in Sierra Leone (11.8%), Mali (12.7%), Ghana (18.1%), Togo (22.0%), Senegal (27.6%), and Liberia (37.9%), but similar to Benin (9.0%) and notably higher than in Burkina Faso (1.9%) (16); These variations may reflect differences in surveillance methods, legal environments, healthcare access, and levels of stigma across countries in the region.

Despite the modest overall decline in HIV prevalence, three of the eight administrative regions reported rates above the national average: Conakry (13.4%), N’Zérékoré (10.5%), and Boké (9.6%). These figures differ from those reported in the 2017 IBBS in Guinea, which documented prevalence rates of 9.1, 17.1, and 12.1%, respectively, in the same regions (14). This variation may be linked to the socio-economic context and the concentration of mining activities in these urban centers, which could influence HIV transmission dynamics.

In this study, HIV prevalence was higher among MSM aged 25 years and older (11.9%) than among those aged 15–24 years (8.0%). This association may reflect longer cumulative exposure to HIV risk factors, including a greater number of sexual partners and unprotected anal intercourse. Notably, while HIV prevalence among older MSM has decreased since the 2017 IBBS study where it was three times higher than in younger MSM (21.9% vs. 7.5%) the prevalence among younger MSM appears to be rising, underscoring the urgent need to focus prevention efforts on this younger age group (14). Similar results were reported by Duah et al. in Sierra Leone in 2021, where HIV prevalence among MSM aged 30 and older was five times higher than among those aged 18–24 years (17).

Our findings also showed that HIV prevalence among homosexual men (12.5%) was approximatively twice that observed among bisexual men (6.4%). While the prevalence in bisexual men is lower, it remains a significant concern given their potential role as a bridge population facilitating HIV transmission to the general population through sexual networks involving both men and women. Notably, these rates are substantially lower than those reported by Loukabou M. et al. in the Democratic Republic of Congo in 2021, where HIV prevalence among bisexual and homosexual men reached 52 and 44%, respectively (18).

Furthermore, three key factors, age, presence of sexually transmitted infections (STIs), and knowledge of a regular partner’s HIV status were significantly associated with HIV infection in this population. MSM aged 25 years and older were approximatively twice as likely to be HIV positive compared to their younger counterparts (OR: 1.78; 95% CI: 1.247–2.539; *p* = 0.001). These findings are consistent with studies by Maria L. et al. in Mali (2020) and Mbongolo L. et al. in the Democratic Republic of Congo (2021), which also identified age ≥25 as an independent risk factor for HIV infection (19, 20).

Similarly, MSM who reported experiencing STI symptoms in the past 6 months had significantly higher odds of HIV seropositivity, reinforcing the well-documented syndemic relationship between HIV and other sexually transmitted infections. This finding aligns with the results reported by Maria L. et al. in Bamako (2018), where STI symptoms were also significantly associated with HIV infection (19).

In our study, MSM who were unaware of their regular partner’s HIV status had a markedly higher HIV prevalence (11.7%) compared to those who were aware (7.9%), with a significantly increased adjusted odds of infection (aOR = 1.60; 95% CI: 1.11–2.31; *p* = 0.012). This association may be a reflection of limited serostatus disclosure and inadequate partner communication, both of which are likely influenced by prevailing stigma, fear of rejection, and punitive legal frameworks criminalizing same-sex sexual behavior in Guinea.

These structural barriers may deter MSM from disclosing their status or inquiring about their partner’s, thus impeding trust and engagement in preventive behaviors. Moreover, fear of discrimination in healthcare settings may contribute to delays in seeking STI treatment or HIV testing, thereby exacerbating transmission risks. Although several high-risk practices such as group sex, oral sex, sex under the influence of substances, and transactional sex were not independently associated with HIV seropositivity in the multivariate model, their high frequency in the study population suggests a potential cumulative risk that warrants further investigation. To better understand these dynamics, a complementary qualitative study is necessary to explore the stigma-related, legal, and human rights barriers that hinder HIV service uptake, testing, and disclosure among MSM in Guinea.

While this study is among the first to evaluate HIV prevalence and associated factors among MSM in Guinea, its findings should be interpreted with caution due to certain limitations, including potential social desirability bias in self-reported sexual behaviors and the cross-sectional study design, which restricts the ability to draw causal inferences.

## Conclusion

This study highlights a persistently high HIV prevalence among men who have sex with men (MSM) in Guinea and identifies several behavioral and demographic factors associated with increased odds of HIV infection. These findings underscore the urgent need for tailored, evidence-informed strategies to strengthen HIV prevention, testing, and care services for this key population. Achieving the UNAIDS 95–95-95 targets will require not only the expansion of MSM-friendly and stigma-free service delivery models, but also concerted efforts to address legal, structural, and social barriers that limit healthcare access and hinder disclosure. Future research should further explore the availability, accessibility, and quality of HIV-related services for MSM, as well as the impact of stigma and criminalization on health-seeking behaviors. Such insights are essential to inform the development of inclusive, rights-based public health programs that effectively reduce HIV transmission and promote equity in care.

## Data Availability

The raw data supporting the conclusions of this article will be made available by the authors, without undue reservation.

## Footnotes

^1^Authors (Unpublished). Guinea_IBBS_Protocol_2022_Unpublished.
